# Long-Distance and Trans-Generational Stomatal Patterning by CO_2_ Across *Arabidopsis* Organs

**DOI:** 10.3389/fpls.2018.01714

**Published:** 2018-11-30

**Authors:** Miranda J. Haus, Mao Li, Daniel H. Chitwood, Thomas W. Jacobs

**Affiliations:** ^1^Department of Plant Biology, University of Illinois at Urbana–Champaign, Urbana, IL, United States; ^2^Department of Plant Biology, Michigan State University, East Lansing, MI, United States; ^3^Donald Danforth Plant Science Center, St. Louis, MO, United States; ^4^Department of Horticulture, Michigan State University, East Lansing, MI, United States; ^5^Department of Computational Mathematics, Science and Engineering, Michigan State University, East Lansing, MI, United States

**Keywords:** satellite stomata, stomatal patterning, long-distance signaling, dual-environment chambers, optical topometry, persistent homology

## Abstract

Stomata control water loss and carbon dioxide uptake by both altering pore aperture and developmental patterning. Stomatal patterning is regulated by environmental factors including atmospheric carbon dioxide (*p*[CO_2_]), which is increasing globally at an unprecedented rate. Mature leaves are known to convey developmental cues to immature leaves in response to *p*[CO_2_], but the developmental mechanisms are unknown. To characterize changes in stomatal patterning resulting from signals moving from mature to developing leaves, we constructed a dual-chamber growth system in which rosette and cauline leaves of *Arabidopsis thaliana* were subjected to differing *p*[CO_2_]. Young rosette tissue was found to adjust stomatal index (SI, the proportion of stomata to total cell number) in response to both the current environment and the environment experienced by mature rosette tissue, whereas cauline leaves appear to be insensitive to *p*[CO_2_] treatment. It is likely that cauline leaves and cotyledons deploy mechanisms for controlling stomatal development that share common but also deploy distinctive mechanisms to that operating in rosette leaves. The effect of *p*[CO_2_] on stomatal development is retained in cotyledons of the next generation, however, this effect does not occur in pre-germination stomatal lineage cells but only after germination. Finally, these data suggest that *p*[CO_2_] affects regulation of stomatal development specifically through the development of satellite stomata (stomata induced by signals from a neighboring stomate) during spacing divisions and not the basal pathway. To our knowledge, this is the first report identifying developmental steps responsible for altered stomatal patterning to *p*[CO_2_] and its trans-generational inheritance.

## Introduction

Stomata are microscopic pores on the plant epidermis whose controlled opening and closing regulates carbon dioxide uptake and transpirational water loss. Each pore is defined by a pair of guard cells whose deformations adjust the pore aperture in response to environmental cues, maximizing water use efficiency. Environmental conditions during leaf development affect the density and distribution of stomata over each leaf’s surface. Both stomatal density (SD, number of stomata per mm^2^) and stomatal index (SI, proportion of stomata per total epidermal cell number) are responsive to the plant’s perception of humidity, light quality and quantity, and *p*[CO_2_] (Woodward and Kelly, 1995; Lake and Woodward, 2008; Casson et al., 2009; Kang et al., 2009; Pillitteri and Torii, 2012; Šantrůček et al., 2014). When we consider the environmental effect of doubling *p*[CO_2_], SI usually decreases by an average 29%, but large variation exists both within and across species such that increased SI is not uncommon (Woodward and Kelly, 1995; Woodward et al., 2002). The inverse correlation between SI and *p*[CO_2_] within indicator species is sufficiently strong that SI in fossilized or dried leaves is a proxy for paleoclimates’ *p*[CO_2_] (McElwain et al., 1999; Beerling and Royer, 2002; Steinthorsdottir et al., 2011).

That said, stomatal development is simultaneously regulated by many environmental cues. A response interaction occurs between water availability (i.e., high humidity, drought, etc.) and *p*[CO_2_] that is genotype specific. In *Arabidopsis*, Col-0 and Ws accessions were grown in elevated *p*[CO_2_] and either well-watered and drought treatments (Woodward et al., 2002). In the well-watered × elevated *p*[CO_2_] condition, Col-0 had slightly reduced SD (∼5%), but had greatly reduced SD in the drought × elevated *p*[CO_2_] condition (∼25%). Ws had strongly reduced SD in both well-watered and drought × elevated *p*[CO_2_] conditions (∼20 and 30%, respectively).

Stomatal patterning is composed of stomatal size, number, and spacing. Transpiration rate is reduced in plants with fewer or smaller stomata, but not for aberrant spacing. Stomatal patterning has a distinct effect on mesophyll patterning, which in turn affects leaf photosynthetic efficiency (Dow et al., 2013, 2014, 2017; Dow and Bergmann, 2014). While previous work has linked stomatal density and clustering to photosynthetic rate, the quantification of stomatal patterning in terms of morphological distribution has not yet been described.

A developmental framework for the molecular control of stomatal patterning is well established for the *Arabidopsis* epidermis. This model provides a template onto which steps sensitive to environmental factors may now be mapped. Stomatal development occurs through a basal pathway in which precursor meristemoid mother cells differentiate into triangular meristemoids. These then differentiate into guard mother cells (GMCs) that finally divide symmetrically to generate the two guard cells that define the stomatal pore.

**FIGURE 1:**
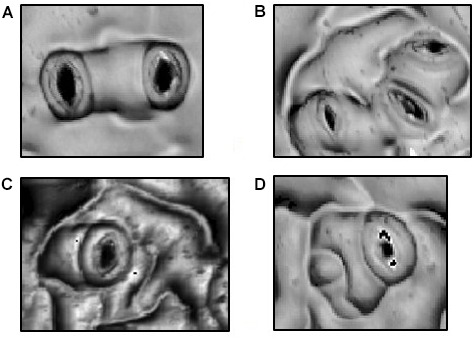
Morphologies in Stomatal Patterning. **(A)** Two satellite stomata resulting from a single spacing division. **(B)** Triplet satellite stomata resulting from two spacing divisions. **(C)** Amplification divisions in which three, successively smaller stomatal lineage ground cells separate the stoma from potential neighboring stomata. **(D)** Combination of a spacing division and a single amplification division.

Superimposed upon the basal pathway, stomatal patterning in *Arabidopsis* adheres to a “One Cell Spacing” rule whereby guard cells of two stomata are never in contact with one another (Figure 1). All stomata are thus surrounded by pavement cells that collaborate in the osmotic reactions that deform guard cells and adjust stomatal pore apertures. Meristemoids can undergo one to three asymmetric divisions to amplify the number of stomatal lineage ground cells (SLGCs), leaving a signature spiral pattern of three adjacent pavement cells of successively smaller sizes (Figure 1C; Delgado et al., 2011). The One Cell Spacing rule is also enforced when a satellite stomate is produced on the opposite side of an SLGC from a previously formed stomate (Figure 1). As in amplification divisions, this division pattern is readily identified where two stomata are separated by a single, unlobed pavement cell.

Forward genetics in *Arabidopsis* has revealed over 35 genes active in the basal stomatal development pathway (Abrash and Bergmann, 2009; Rowe and Bergmann, 2010; Pillitteri and Torii, 2012; Pillitteri and Dong, 2013; Engineer et al., 2014). Genes regulating the pathway’s sensitivity to environmental conditions have also begun to emerge. The first gene identified to connect *p*[CO_2_] to stomatal development was *HIGH CARBON DIOXIDE* (*HIC*), a putative 3-keto acyl coenzyme A synthase expressed in guard cells (Gray et al., 2000). *hic* mutants display an increase in SI in response to elevated *p*[CO_2_], but the mechanism of *HIC*’s role in the plants’ SI phenotype has yet to be elucidated.

*EPIDERMAL PATTERNING FACTOR2 (EPF2)* and *CO_2_ RESPONSE SECRETED PROTEIN (CRSP)* encode secretory proteins that play a role in *p*[CO_2_] regulation of stomatal development (Hu et al., 2010; Doheny-Adams et al., 2012; Engineer et al., 2014). CRSP is a member of the subtilisin-like family of secretory proteases that cleave transmembrane proteins (Engineer et al., 2014). EPF2 is an extracellular protein that inhibits stomatal development and binds CRSP *in vitro*. When grown in elevated *p*[CO_2_], loss-of-function *epf2* or *crsp* mutants display a higher (rather than typically reported lower) SI than do plants grown in ambient *p*[CO_2_]. In addition to these proteins, the β-carbonic anhydrase and the hormone abscisic acid (ABA) regulate the changes in both stomatal physiology and development in response to changes in the *p*[CO_2_] (Chater et al., 2014, 2015; Engineer et al., 2014; Qi and Torii, 2018). Carbonic anhydrases catalyze the conversion of water and CO_2_ into bicarbonate. When both *CARBONIC ANHYDRASE1* and *4 (CA1, CA4)* are inactivated, SI also increases under elevated *p*[CO_2_] (Engineer et al., 2014). *CRSP* mRNA levels decrease in elevated *p*[CO_2_], but both *CRSP* and *EPF2* mRNA levels increase under elevated *p*[CO_2_] in *ca1ca4* double mutants. Yet the enzymatic mechanisms by which CA1 and CA4 affect the stomatal development pathway are unknown. ABA mediates changes in stomatal density via reactive oxygen species such that ABA mutant lines have higher stomatal density (Chater et al., 2015). The ABA mutant lines can not alter stomatal density in elevated *p*[CO_2_]. The regulation of stomatal physiology and development by relative humidity and *p*[CO_2_] are linked through ABA. Stomatal density increases in elevated relative humidity conditions for a variety of species including *Arabidopsis*, cucumber, tomato, sweet pepper, eggplant, and rose (Bakker, 1991; Torre et al., 2003; Arve et al., 2015).

Long distance communication networks in plants have evolved to relay environmental perceptions from mature organs to developing tissue nearer to meristems. This quintessentially botanical developmental strategy allows newly developed organs to make developmental adjustments “on the fly,” as light quality and quantity, water and nutrient availability and even *p*[CO_2_] change during a single life cycle (Lough and Lucas, 2006; Wigge, 2011; Uchida et al., 2012; Warschefsky et al., 2016). A variety of macromolecules, including mRNAs, small RNAs, small proteins, and diverse small metabolites, mediate this phloem-borne long distance signaling. The plasticity of stomatal development in response to environment is a likely candidate for just this type of inter-organ communication. Indeed, mature *Arabidopsis* rosette leaves have been shown to influence, presumably via mobile molecular cues, the development of emerging leaves in response to light intensity and *p*[CO_2_] experienced by the former (Lake et al., 2001). In addition, stomatal conductance of older leaves is strongly correlated with SI of younger leaves in poplar (*Populus trichocarpa × P. deltoids*) (Miyazawa et al., 2006). In spite of evidence for the existence of this mobile signal, the agent(s) of long-distance communication of *p*[CO_2_] for stomatal development remain unknown.

Many advances in identifying genetic and biochemical pathways through which *p*[CO_2_] affects stomatal development, but the resulting changes in stomatal patterning remain incompletely described. An aim of this study was to more precisely characterize variation in the stomatal development pathways under differing *p*[CO_2_] regimes by analyzing differences in stomatal patterning both through the basal pathway and through subsequent spacing divisions. Our second objective was to extend the previous longitudinal study (Lake et al., 2001) of *p*[CO_2_] inter-organ stomatal developmental signaling and assess whether such signaling extends across generations from mother plant to seed. To these ends, we constructed a dual-chamber plant growth system that allowed us to subject emergent and mature rosette and cauline leaves to differing *p*[CO_2_]. The system allowed us to characterize the effects on stomatal patterning of differential gaseous environments experienced by rosette and cauline leaves of *Arabidopsis*.

## Materials and Methods

### Plant Growth in Dual-Environment Chambers

**FIGURE 2:**
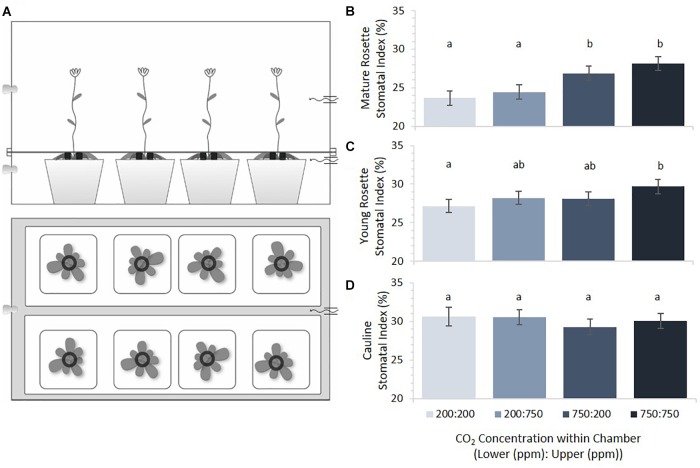
Stomatal indices across plant tissues as a function of [CO_2_]. **(A)** Dual-compartment bench top chamber design. Each chamber had two rows of four 5.5 cm^2^ pots. **(B)** Mature rosette leaf stomatal index under four CO_2_ combinations (error bars, s.e.m.; *n* = 16). **(C)** Young rosette leaf stomatal index under four CO_2_ combinations. SI of mature and young rosette leaves in 200 ppm was significantly different from those in 750 ppm (*p* < 0.0002, *p* = 0.02, respectively). **(D)** Cauline leaves under four CO_2_ combinations. Cauline leaf SI did not significantly differ with [CO_2_] (*p* = 0.68). Letters indicate similarity based on a *p*-value of 0.05 for independent pair-wise comparisons.

Dual compartment bench-top, polycarbonate growth chambers were constructed in order to subject apical and basal portions of *Arabidopsis thaliana* plants to differential gaseous environments (Figure 2A). The lower compartment contained the mature rosette and the upper compartment contained younger rosette leaves and cauline leaves of the inflorescence. *Arabidopsis* accession Col-0 seeds were imbibed and stratified for 3 days to synchronize germination before planting. Seeds were planted in pots of 9 × 4 Compak^TM^ trays containing a 3:1 (vol:vol) mixture of Sunshine LC1^TM^: medium Vermiculite (approximately 100 cm^3^ of soil/plant), pre-wetted with a 1.2 g/L Gnatrol^TM^/water solution to control fungus gnat (*Bradysia coprophila*) infestation. Eight pots were placed in each of four plastic bench top dual- compartment chambers supplied with a continual flow of two component pre-mixed CO_2_ + balanced air cylindrical tanks charged with *p*[CO_2_] at either 200ppm or 750ppm (S. J. Smith, Champaign, IL, United States). Premixed gas entered the chamber compartments through 1/4 ID Nalgene tubing at a rate of 4 scfh (Dwyer Model VFA-1-BV, Visi-float^®^ flowmeters). Actual *p*[CO_2_] was measured with gas sensors (Vernier Model CO2-BTA) to be slightly higher than expected, at approximately 325 and 850 ppm, likely due to condensation from high humidity inside the chambers. Growth conditions consisted of 12 h days/12 h nights at 18–20°C under 48^′′^ T8 cool-white, high output fluorescent lamps (32W, 4100°K). Data loggers (Onset HOBO^®^ Model U12-012) monitored temperature, relative humidity, and light levels (Supplementary Figure 4). Two weeks after planting, each pot was thinned to one plant.

After 4 weeks, when leaf six was fully expanded (about 50% rosette growth according to Boyes, 2001) and plants were still in visibly vegetative phase producing new rosette leaves, chambers were converted from single bench top chambers to dual- compartment chambers. The plastic divider separating upper and lower compartments bore eight 1.5 cm holes, each lined with 1.24 cm poly natural foam collars in order to separate and seal older rosette leaves from younger rosette and cauline leaves. The junction between the upper and lower halves of each chamber was sealed using gaffer’s tape (Tecnec^®^).

After inflorescences had bolted (mid-flowering; Boyes, 2001), optical topometry (OT) abaxial measurements were taken from the first cauline leaf of the inflorescence, the youngest 1 cm rosette leaf to develop in the chamber’s upper half and leaf 6 from the mature rosette in the lower half. All measurements were taken with a Nanofocus μsurf explorer^®^ optical topometer at 50× magnification with a 0.8 numerical aperture. Leaf epicuticular wax accumulation was estimated using the reflective intensity of an optical topometry surface measurement (Haus et al., 2015). Stomata and pavement cells were counted with ImageJ software cell counter tool. SI is presented in text and SD can be found in Supplementary Figure 1.

### Transgenerational *p*[CO_2_] Effects

Col-0 seeds that matured on parental plants grown in either 400 or 1500 ppm *p*[CO_2_] for two generations were imbibed in 50% glycerol for 3 days. Ungerminated cotyledons were separated from seed coats and radicles. OT measurements were taken on the abaxial side of the cotyledons as described above. Reflective intensity surfaces from OT measurements were used to enumerate stomatal precursors (identifiable meristemoids and GMCs) and pavement cells on the abaxial side of all samples. Stomatal Precursor Index (SPI) was calculated as the quotient of the number of stomatal precursor cells by total epidermal cell number (pavement and stomatal precursors).

Col-0 seeds that had matured in either 400 or 1500 ppm *p*[CO_2_] were also germinated in both 400 and 1500 ppm *p*[CO_2_], resulting in four atmospheric treatment combinations. Abaxial cotyledon surfaces from seeds matured under each of these treatment regimes were measured using OT as described above. Pavement cell number, pavement cell size, stomate number, satellite stomate number (Figures 1A, D) and triplet stomata numbers (Figure 1B) were obtained and satellite index (SatI) was calculated as the proportion of satellite and amplification events over the total number of stomata. The coordinates (*x*, *y*) of each stomate was recorded and used to derive a mean distance between each possible stomatal pair. Pavement cell size measured only cells completely enclosed within the image or with partial single lobe missing.

### Statistical Analyses

In order to determine the effect of stomatal and pavement cell patterning to differential *p*[CO_2_] across plant organs, a two-way ANOVA was run in SAS PROC MIXED. The experimental model was as follows:

$$Y_{ijk}=\mu +M_{i}+D_{j}+{\mathrm{MD}}_{ij}+e_{\left(\mathrm{ij}\right)k}$$

Where *Y_ijk_* represents the phenotypic mean of a genotype, M_i_ is the effect of the ith *p*[CO_2_] experienced by mature tissue, D_j_ is the effect of the jth *p*[CO_2_] experienced by the developing tissue, MD_ij_ is the interaction of the ith *p*[CO_2_] experienced by mature tissue and jth *p*[CO_2_] experienced by the developing tissue, and e_ijk_ represents the residual error. All effects were considered fixed. This model was applied to both the dual-chamber leaf tissue and trans-generational cotyledon experiments. In the dual-chamber leaf tissue experiment, the mature tissue’s gaseous environment was considered to be in the lower chamber (M_i_) and the developing tissue’s gaseous environment was considered to be in the upper chamber (D_j_). In addition to effect significance, pairwise comparisons were calculated for each treatment combination. Leaf type (mature rosette, young rosette, and cauline) had a strong effect in the model, so each was analyzed independently. In the trans-generational cotyledon study, the mature tissue’s gaseous environment was considered to be that experienced by the parental plant (M_i_) and the developing tissue’s atmospheric environment was considered to be that experienced by the germinating cotyledon (D_j_).

### A Persistent Homology Based Method Quantifies Stomatal Distribution

A persistent homology based method was employed to describe stomatal patterning on cotyledons from seed set in either 400 or 1500 ppm *p*[CO_2_] and then germinated in either 400 or 1500 ppm *p*[CO_2_]. Using the coordinates from each stomate, Betti0 (the number of connected components) curves were calculated for each image. To determine statistical significance between treatments, 10,000 random permutations were run and the mean Betti0 curve was calculated for each group (4 curves each permutation). Then the pairwise distance among those four treatments was calculated from which the L_2_ norm of the pairwise distance was determined and used to determine a *p*-value for significance. Further details of this method are provided in Results.

## Results

### Long-Distance Signaling in Stomatal Development Across Leaf Types

Previous studies had suggested that mature rosette leaves of *Arabidopsis* perceive environmental conditions and transduce those perceptions into a mobile signal that influences stomatal density and index in subsequently emerging leaves (Lake et al., 2001; Coupe et al., 2006). Within these studies, however, the distance between rosette leaves and developing leaves was short (three plastochrons) and the meristem may have residual information from the mature tissues’ environment. We therefore constructed a dual environment growth system in order to study whether signaling between rosette leaves and emerging leaves show the same effect with both a greater distance (i.e., younger leaves) and an anatomically different path (i.e., cauline leaves). Our two-compartment chamber systems exposed mature rosette leaves to distinct atmospheric treatments from that experienced by young rosette and cauline leaves on the same plant (Figure 2A). Mature rosette leaves were subjected to the *p*[CO_2_] of the lower chamber, while young rosette and cauline leaves experienced a separately regulated *p*[CO_2_] in the upper chamber. Using this system, we set out to test the hypothesis that a mobile signal from mature leaves directs stomatal development in emerging leaves based on the *p*[CO_2_] of the formers’ environment.

Mature rosette, young rosette, and cauline leaves from plants grown in each of four possible combinations of 200 and 750 ppm *p*[CO_2_] in upper/lower chamber compartments were scored for SI (Figure 2B). Results are ordered such that the lower chamber *p*[CO_2_] is presented first (Lower:Upper). Within the same treatment combinations, cauline leaves had the highest SI, followed by young rosette leaves, with mature rosette leaves having the lowest SI. This difference was significant (*p* < 0.0001) and highlights developmentally distinct control such that younger leaves always have a larger SI than mature leaves. Overall, *Arabidopsis* rosette leaves developing in this system responded to higher *p*[CO_2_] by increasing, not decreasing, SI. Mature leaves responded the most dramatically, with a significant increase in SI when grown at elevated *p*[CO_2_] compared to growth at sub-ambient levels (Figure 2B, *p* = 0.0002). Mature leaves were not significantly responsive to the *p*[CO_2_] experienced by younger leaves emerging acropetally to them (*p* = 0.25).

Emerging rosette leaves displayed a pattern of responses to *p*[CO_2_] similar to that of mature leaves, but the effect was less dramatic, with a statistically significant difference only between the 200/200 and 750/750 treatments (Figure 2C, *p* = 0.02). When mature and young rosette leaves on the same plants experienced reciprocal *p*[CO_2_] treatments, the SI of young leaves was intermediate between that of developmentally similar leaves on plants grown under uniformly high or low *p*[CO_2_] conditions. The two intermediate indices did not differ from one another (Figure 2C, *p* = 0.92), nor from either uniform *p*[CO_2_] treatment. The SI of developing young rosette leaves was affected by both the *p*[CO_2_] that they experienced and that mature leaves had experienced, but not significantly (*p* = 0.08 and 0.11, respectively). These results differ slightly from those reported in previous studies where young leaves were significantly affected by conditions experienced by leaves emerging before them (Lake et al., 2001; Coupe et al., 2006). Interestingly, cauline leaves’ SI appears to be strikingly impervious to variation in the *p*[CO_2_] that they, or organs emerging before them, experience (Figure 2D, *p* = 0.6745).

### Trans-Generational Signaling in Stomatal Development

Overall, the effect of basipetal *p*[CO_2_] influence on developing tissue is reduced with greater developmental distance, and completely disappears in cauline leaves. This could be related to shifts in developmental priorities of sink tissue. If such signals are stable but dependent upon sink strength, then they may still be transmitted trans-generationally, from the mother plant to the cotyledons of the next generation’s seeds. To test this possibility, seeds that matured on plants grown in 400 or 1500 ppm *p*[CO_2_] were collected and germinated in either 400 or 1500 ppm *p*[CO_2_], yielding four treatments: 400:400, 400:1500, 1500:400, and 1500:1500 ppm (Parental:Germinated). Germinating cotyledons from each regime were scored for SI when they had reached a minimum of 3 mm and were no longer expanding.

**FIGURE 3:**
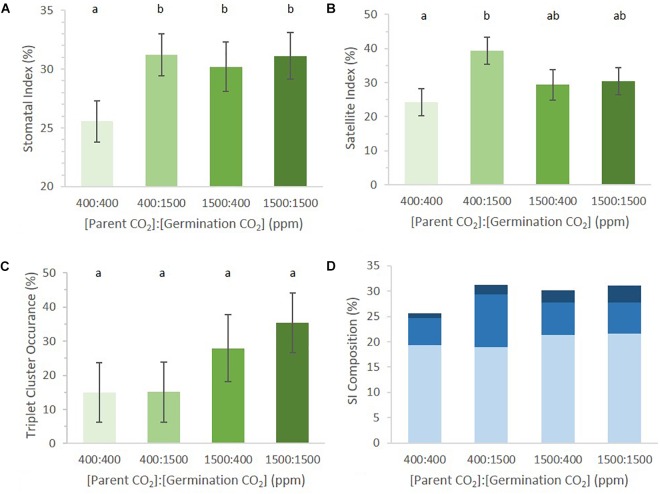
Abaxial stomatal patterning in cotyledons from seed set and germinated in differing p[CO_2_] environments (error bars, s.e.m.; *n* = 10). **(A)** SI was greater in all conditions where p[CO_2_] was elevated (*p* = 0.04). **(B)** SatI across the four treatments. **(C)** Proportion of satellite stomata that made a third satellite was higher in 1500 ppm parental environment than 400 ppm parental environment (*p* = 0.076). **(D)** Composition breakdown of all stomata. Light blue indicates percentage basal-pathway stomata, medium blue indicates percentage satellite stomata, and dark blue indicates percentage triplet satellite stomata. Letters indicate similarity based on a *p*-value of 0.05 for independent pair-wise comparisons.

SI of germinating cotyledons was significantly lower in the 400:400 ppm treatment than in any regime that included exposure to 1500 ppm *p*[CO_2_] (Figure 3A, *p* = 0.04). It did not matter whether cotyledons were exposed to 1500 ppm *p*[CO_2_] while maturing in the mother plant or during germination for SI to be higher than that in 400:400 ppm treated material. Seedlings that received a 1500 ppm *p*[CO_2_] treatment did not differ from one another.

**FIGURE 4:**
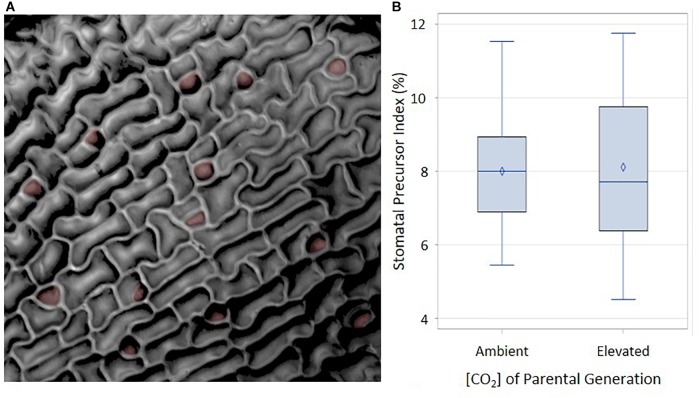
Abaxial distribution of SPI in ambient and elevated maternal conditions. **(A)** Reflective intensity of the surface of the abaxial side of a cotyledon. Stomatal precursors are false colored in red. **(B)** Using an α of 0.05, there was no significant difference between ambient and elevated maternal [CO_2_]. *F* = 0.02 and *p*-value of 0.9004.

A possible mechanism mediating the difference in cotyledon SI at ambient and elevated *p*[CO_2_] is that the organs developing in the seed at 1500 ppm produce a higher proportion of stomatal precursors than those developing at 400 ppm. Cotyledons from ungerminated seeds from these two treatments would be expected to display stomatal differences in the epidermis prior to germination, since stomatal meristemoids and GMCs begin to develop during seed maturation (Geisler and Sack, 2002). Additional stomata or precursors would then differentiate from this basal set of progenitor cells during germination. If the stomatal precursor index (SPI, proportion of meristemoids and GMCs to total cell number) is determined by the parental environment during seed development and if parental *p*[CO_2_] influences cotyledon stomatal development, then SPI of ungerminated cotyledons should be seen to vary in response to *p*[CO_2_]. We grew plants and collected seed for two generations in either 400 ppm or 1500 ppm. Resulting seeds were dissected and ungerminated cotyledons were scored for SPI (Geisler and Sack, 2002; Delgado et al., 2011). Immature cotyledons from each parental plant begin with the same proportion of stomatal precursor cells per total cell number regardless of *p*[CO_2_], although the distribution is wider under the elevated *p*[CO_2_] treatment (Figure 4). These data suggest that the environment of the parental plant does not affect stomatal development during the earliest stages of cotyledon development. Any affect that the parental environment has on the final cotyledon stomatal population must therefore be imposed after the initial cotyledon stomatal population is established, perhaps via subsequent amplification divisions or the generation of new stomatal precursors.

To address this possibility, we revisited the previous data set, scoring for stereotypical signatures of stomatal division patterning (Figure 1). During epidermal development, cells will undergo waves of cell division and expansion, leaving signatures of patterning (Geisler and Sack, 2002). The more waves the tissue undergoes, the more difficult it becomes to see these signatures as cell expansion can mask division. Cotyledons are excellent organs in which to measure stomatal patterning since they only undergo few waves of cell division and expansion compared to the larger rosette counterparts. For each dissected cotyledon, we calculated satellite index (SatI, proportion of satellite stomata per total stomata, Figure 1A) and the proportion of triplet stomata per total satellite stomata (Figure 1B). Amplification events (Figure 1C) were rare and thus were not included.

The germination environment was found to play a greater, albeit non-significant, role in determining SatI than the parental environment (germination environment *p* = 0.06, parental environment *p* = 0.65). There was no significant difference in SatI between different parental environments within the same germinating environment. However, an interesting trend emerged between cotyledons from identical parental environments germinating in different environments. SatI was highest in 400:1500 ppm cotyledons and lowest in 400:400 ppm cotyledons (Figure 3B, *p* = 0.01). Parental 1500 ppm cotyledons displayed statistically identical SatI under differing germinating environments. These results support the hypothesis that formation of satellite stomata is one point of developmental regulation by *p*[CO_2_] during stomatal development.

The number of satellite stomata that proceeded to produce an additional triplet of stomata did not differ among the four treatments (Figure 3C, *p* = 0.68). Among satellite stomata formed on 1500:1500 ppm cotyledons, 35% went on to create a new satellite stomate compared to 28% in 1500:400 ppm cotyledons. Only 15% of satellites in 400:400 and 1500:400 ppm cotyledons created an additional satellite. The parental environment may regulate triplet satellite formation, although the effect was not statistically significant (Figure 3C, *p* = 0.076).

The proportions of stomata arising from the basal pathway vs. satellite divisions in cotyledons of seeds borne by parental plants grown in 400 and 1500 environments were not significantly different (approximately 19 and 21% in 400 and 1500 parental environments, respectively, Figure 3D). However, differences in satellite division patterning collectively result in changes in SI. This is an example of small changes in individual phenes (SatI and triplet occurrence) collectively contributing to a change in an aggregate quantitative phenotype such as SI (Lynch, 2015).

### Changes in Stomatal Distribution

Previous reports have noted that stomatal distribution can affect leaf morphological phenotypes such as mesophyll patterning and, ultimately, photosynthetic efficiency (Dow et al., 2013, 2017; Dow and Bergmann, 2014). We observed that, while stomata were rarely clustered in the traditional sense (two stomata lacking a spacer pavement cell, breaking the one-cell-spacing rule), there were yet instances of aberrant stomatal distribution in some treatments. For example, ten stomata may be uniformly dispersed across the epidermis (Supplementary Figure 2A). Alternatively, they may be less unevenly dispersed, for example in groups of three (Supplementary Figure 2B). We set out to determine if stomatal distribution was quantifiably different when grown in various *p*[CO_2_].

**FIGURE 5:**
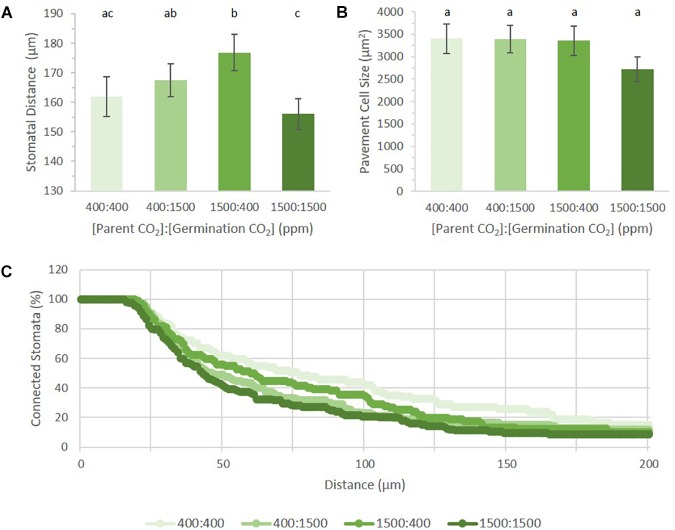
Stomatal distribution on cotyledons from seed set and germinated in differing *p*[CO_2_] environments (error bars, s.e.m.; *n* = 10). **(A)** Average distance between two stomata across four treatments. **(B)** Average pavement cell size in four treatments. **(C)** Persistent homology based method describing distribution of stomata across the image. Individual treatment combinations are significant (*p* = 0.03). Letters indicate similarity based on a *p*-value of 0.05 for independent pair-wise comparisons.

Among the four treatments, the mean distance between proximate stomata differed significantly (*p* = 0.0012, Figure 5A). Given that SatI was highest in 400:1500 ppm cotyledons (Figures 3B,D), it follows that these cotyledons should display the shortest mean distance between proximate stomata. Instead, the 1500:1500 ppm treatment displayed the shortest mean stomatal distance, potentially due to its having both a high SI and higher triplet proportion (Figures 3A,C). However, cotyledons from the 1500:400 treatment show the same stomatal patterning as the 1500:1500 treatment, yet had the greatest mean stomatal difference (176.8 μm, *p* = 0.02). This discrepancy could also be explained if pavement cell size differed among treatments, but no significant difference in pavement cell sizes was observed (Figure 5B). Neither were these results readily rationalized from SI, SatI or triplet data. This inconsistency led us to pursue alternative methods for quantifying stomatal distribution.

Topological Data Analysis (TDA) is used to quantify shape and structure in data. Within TDA, persistent homology is a mathematical tool used to track topological features across a function, or filtration (Li et al., 2018). One of the primary units used to describe topological space is the Betti number, where the *nth* Betti number (β_n_) describes the connectivity of topological features such as connected components or blobs (*n* = 0), holes (*n* = 1), and voids (*n* = 2). In analyzing our data, we used zeroth Betti number (β_0_) values to describe the distribution of points (stomatal pores) relative to one another by their incorporation into a connected component (blob) as a function of their radius. Each stomate is assigned a point as an independent component. A radius expands from this point until it interacts with the radius from another stomate. The two stomata then collapse into a single connected component (Li et al., 2018). The rate of point collapse into a single connected component can then be compared across treatments as a curve, plotting the number of connected components as a function of radius length (Figure 5C).

A linear pattern (or a steady, stepwise slope) indicates a random distribution, while intermittent or steep changes in slopes indicate non-random distribution, or groupings, with respect to stomatal distances from each other (near or far). When applied to our dataset, we can see that each treatment produced a distinct pattern of incorporation (*p* = 0.03). Figure 5C shows the percentage of connected stomata to total stomata per image. Raw values can be seen in Supplementary Figure 2C. Mature *Arabidopsis* stomata have a radius of 10–15 μm. In this analysis, a reference point was placed at the center of each stomatal pore. The number of connected components does not change until the radius has passed this 10–15 μm threshold, generating an initial plateau for each treatment. Although the field of view is 320 μm, all stomata were connected by a 200 μm radius for all treatments. Plants grown in 400:400 had the steadiest rate of stomatal incorporation, while plants grown in 1500:1500 had an initial sharp drop followed by a steady rate of incorporation after 100 μm. Principal component analysis on the dataset shows the first (PC1) and second component (PC2) capture 73.08 and 13.61% of the variation, respectively (Supplementary Figure 2D). PC1 has high values within the first 50 μm, implying the initial number of stomata and the number of closer stomata occupy the most variance (Supplementary Figure 2E). PC2 loads are high in the middle (around 70–120 μm), suggesting accountable variance is also present at a wider scale of stomatal patterning in addition to that found by PC1.

### Epicuticular Wax Accumulation

The amount and composition of epicuticular wax on plant surfaces is sensitive to *p*[CO_2_] (Bird and Gray, 2003). Several epicuticular wax biosynthesis genes have been reported to govern the SI response to *p*[CO_2_] (Bird and Gray, 2003). Therefore, epicuticular wax may be physiologically involved in mobile signaling that controls stomatal numbers. We estimated cuticular wax levels from plants grown in the bench top dual-environment chambers using optical topometry (OT) (Haus et al., 2015). The results of this study were strikingly consistent: regardless of leaf type, *p*[CO_2_] of the upper chamber (younger leaves) dictated wax levels across all three leaf types measured: mature rosette, young rosette and cauline leaves (Supplementary Figure 3A, *p* < 0.0001). Young rosette and cauline leaves responded to *p*[CO_2_] by producing more wax in 750 ppm and less wax in 200 ppm (young rosette leaves *p* = 0.0024; cauline leaves *p* = 0.0097). Interestingly, wax levels on mature rosette leaves appear to correspond with the environment experienced by organs that develop subsequent to them (*p* = 0.0193).

Epicuticular wax levels on the epidermis of cotyledons were also estimated by OT (Supplementary Figure 3B). Cotyledons that experienced only ambient *p*[CO_2_] accumulated significantly less epicuticular wax than those that experienced only elevated *p*[CO_2_] (*p* = 0.0089). As in the dual-chamber experiment described above, wax accumulation corresponded to the current environment (*p* = 0.0088) and significantly less to the parental environment (*p* = 0.31).

## Discussion

Long distance communication networks allow plants to alter development and morphology of young tissue in response to the environment experienced by mature tissue. This adaptation allows newly developed organs to alter development for optimized growth under changing environmental conditions (Lake et al., 2001; Lough and Lucas, 2006). Emergent leaves of *Arabidopsis* plants have been reported to alter stomatal patterning (SD and SI) in response to *p*[CO_2_] levels experienced in mature tissues, but the developmental separation between the two leaf types was only three plastochrons (Lake et al., 2001). In that study, leaf 13 was the last leaf considered “mature tissue” and leaf 16 was collected as “young tissue.” One objective of our study was to examine this phenomenon when a greater developmental interval separated the leaf pairs whose responses were being compared (leaf 6 and youngest emergent leaf >1 cm). Under the environmental conditions presented in our study, we did not find evidence to confirm these reports. The *p*[CO_2_] of mature tissue showed little effect on younger tissues further removed developmentally from the signaling source. Furthermore, the SI of cauline leaves were unresponsive to variation in *p*[CO_2_] that they or rosette leaves experience. Taken together, these results suggest that any mobile signal generated in response to *p*[CO_2_] is not extensively systemic, fading beyond a few rosette leaves. Alternatively, cauline leaf maturation may be governed by a fundamentally different developmental physiology than that of rosette leaves, rendering them impervious to some signals to which the latter readily respond. One plausible explanation could be that ABA and *p*[CO_2_] sensitivity decrease with developmental age, though the extent of this sensitivity in cauline leaves is unknown (Pantin et al., 2013; Chater et al., 2014). Unexpectedly, cotyledon stomatal numbers of germinating *Arabidopsis* seeds were shown to be developmentally plastic and significantly responsive to the *p*[CO_2_] experienced by the parental plant. Information was passed from the 1500 ppm *p*[CO_2_] maternal environment that elicited an increase in SI when seeds were germinated in 400 ppm *p*[CO_2_] (Figure 5A).

We also sought to identify the particular steps of the stomatal development pathway that are sensitive to *p*[CO_2_]. In order to address this question, we needed to identify and quantify key sub-patterns in the epidermis that collectively comprise SI, or specific phenes that contribute to the aggregate SI phenotype. Stomatal development in cotyledons has been observed to occur in synchronous waves of cell division (Geisler and Sack, 2002; Delgado et al., 2011), providing a means to dynamically regulate stomatal patterning throughout growth and development. We provide here a previously unrecognized mechanism for *p*[CO_2_] regulation of the stomatal developmental pathway, that being through the formation of satellite meristemoids (Figure 5). Ungerminated cotyledons present no difference in SPI according to the *p*[CO_2_] of the parental environment (Figure 4), but stomatal lineage analysis reveals unique stomatal composition and distribution in mature cotyledons. When germinated in ambient *p*[CO_2_], seeds that had matured under elevated *p*[CO_2_] displayed a significantly higher stomatal index than those that had matured under ambient *p*[CO_2_].

Genes that play a role in regulating the epidermal developmental response to *p*[CO_2_] have been identified (Gray et al., 2000; Hu et al., 2010; Engineer et al., 2014). Adding to that evolving narrative of environmental control of stomatal development, this study provides the nuance that such *p*[CO_2_] regulation occurs via the formation of additional satellite stomata and not through the basal stomatal development pathway. We have provided support for this hypothesis by identifying phenes that contribute to the aggregate SI phenotype and by applying topological data analyses to characterize subtle relationships within the pattern of stomatal spacing relative to one another.

Additional environmental factors impinge upon the developmental trajectory of the plant epidermis, and these may have overridden the effects of treatments imposed here. For example, the SI of expanding leaves increased under elevated *p*[CO_2_] rather than decreased as previously reported. The disparity between our results and those previously published could be attributable to the very high humidity that developed inside our closed chambers (Supplementary Figure 4). Elevated relative humidity increases stomatal density on many plant species, including *Arabidopsis* (Bakker, 1991; Torre et al., 2003; Arve et al., 2015). The control of stomatal patterning by ABA or *p*[CO_2_] is desensitized by the elevated relative humidity within the bench-top chambers. This hypothesis is supported by previous studies indicating elevated humidity alters localized and systemic signaling of ABA and *p*[CO_2_] stomatal development and physiology (Raschke, 1975; Frechilla, 2002; Talbott et al., 2003; Aliniaeifard and Van Meeteren, 2013; Pantin et al., 2013). It has been reported that the SI response to doubling *p*[CO_2_] concentrations is co-dependent upon water availability (Woodward et al., 2002; Carins Murphy et al., 2014). When grown in elevated *p*[CO_2_], Col-0 *Arabidopsis* plants display a 6% decrease in SI under conditions of normal humidity, but more than a threefold greater decrease in SI under drought conditions (Woodward et al., 2002). If reducing SI under low humidity is an adaptation that evolved to minimize water loss, then increasing SI would be advantageous and under higher humidity. The lack of pressure to reduce water loss may explain why emergent leaves were not seen to reduce SI as profoundly as previously reported. These results highlight the need to study combinatorial stressors in order to determine true effects of climate change impacts (Shaar-Moshe et al., 2017).

Studies on the role of sugar in stomatal patterning suggest that exogenous provision of sucrose, fructose, or glucose (but not mannitol) elicits an increase of stomatal clustering in *Arabidopsis* seedlings grown *in vitro* (Akita et al., 2013). Stomatal clustering results from a disruption of the one-cell-spacing rule that is regulated by spacing and amplification divisions. Sugar treatment may ectopically induce expression of stomatal lineage genes in non-stomatal lineage cells. Similarly, *Alocasia amazonica* plantlets display increased SD when grown in a closed container and fed exogenous sucrose (Jo et al., 2009). Given that carbon resources are stored in unusable forms prior to germination, the difference between available carbon post-germination, either in the atmosphere or stored in the seed, may be the regulating signal defining stomatal development. Post-germinative stomatal development may be combinatorially responsive to signaling from sugars released from stored reserves during germination and ambient *p*[CO_2_].

The chemical nature of the signal controlling stomatal development’s environmental sensitivity is as yet unknown, but may directly or indirectly extend to cuticular wax biosynthesis. Plants carrying mutations in cuticular wax biosynthesis genes display alterations in SI in response to *p*[CO_2_] and other stressors. Thus, cuticular wax may be involved in mobile signals that control stomatal numbers (Gray et al., 2000; Bird and Gray, 2003; Chen et al., 2003; Bourdenx et al., 2011; Yang et al., 2011; Zhou et al., 2015). We observed that cuticular wax accumulation responds to the environment experienced by developing tissue. Alternatively, cuticular wax accumulation may not be involved in developmental signaling, but may simply act as a sink for excess photosynthate. Limited information is available regarding changes in wax composition of leaves throughout the life history of plants. One study followed wax composition over the development of a single leaf (Jetter and Schäffer, 2001). Others have characterized the final outcome of wax deposition in response to environmental cues (Hatterman-Valenti et al., 2011; Yang et al., 2011; Zhou et al., 2015). Our study raises new questions regarding the change of wax composition and deposition as the plant experiences environmental changes throughout its lifetime.

## Author Contributions

MH and TJ conceived the study’s idea. MH designed and carried out the experiments, worked primarily with sample collection, microscopy, and image analyses, and wrote the manuscript. MH and ML analyzed the statistics and designed the figures. ML analyzed the stomatal distribution using persistent homology. TJ, ML, and DC edited the manuscript. All authors discussed the interpretation of the results and contributed to the final manuscript.

## Conflict of Interest Statement

The authors declare that the research was conducted in the absence of any commercial or financial relationships that could be construed as a potential conflict of interest.
