# Supplementary material for: Long-Distance and Trans-Generational Stomatal Patterning by CO2 Across Arabidopsis Organs
Source: Front Plant Sci. 2018 Nov 30;9:1714. doi: 10.3389/fpls.2018.01714 (PMC6287203; doi:10.3389/fpls.2018.01714)
Supplement: Supplementary file 1 [file Data_Sheet_1.PDF]

Supplementary Material

Long-distance and trans-generational stomatal patterning by CO_2 across *Arabidopsis* organs

Miranda Haus*, Mao Li, Dan Chitwood, Tom W. Jacobs

*** Correspondence:** Miranda J. Haus: hausmira@msu.edu

1 Supplementary Figures and Tables

Supplementary figure 1 shows stomatal density, an additional indicator of changes in leaf growth and development, for the dual-chamber and trans-generational experiments.

Supplementary figure 2 shows examples of stomatal distribution (A and B) and Betti0 curves for stomatal number incorporation (C), pavement cell number incorporation (D), and pavement cell incorporation as a percentage (E).

Supplementary figure 3 demonstrates wax deposition for the experiment. Using optical topometry, reflective intensity of epidermal surfaces has been shown to estimate wax quantity. Here, we used it for both the dual-chamber and trans-generational experiments.

Supplementary figure 4 shows sample output from HOBO data logger during the experiment.

1.1 Supplementary Figures

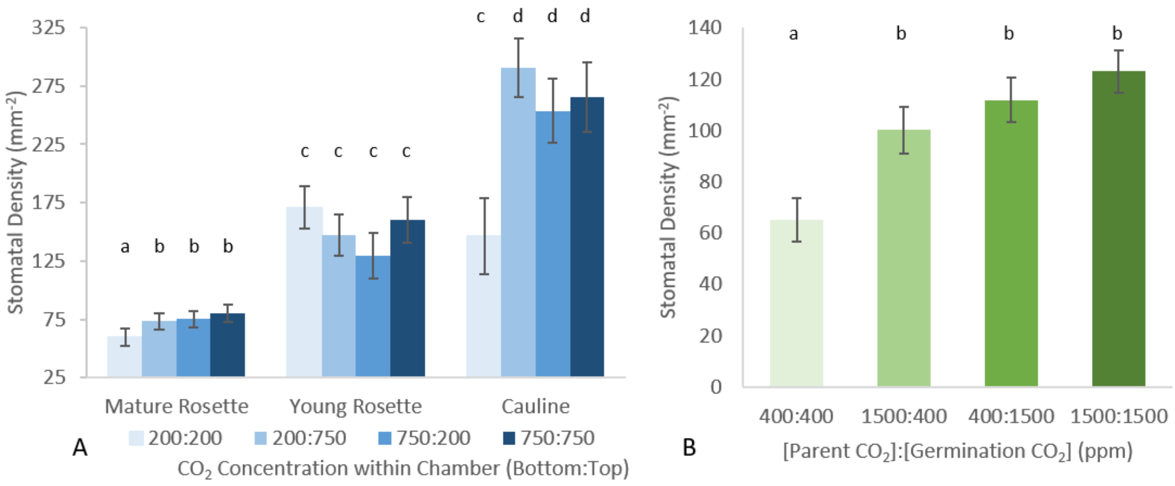

Supplemental Figure 1. Stomatal Density of A. Leaves at three stages from plants grown in a split-environment chamber (error bars, s.e.m.; n = 16. and B. Cotyledons exposed to identical or contrasting parental and germination $p[CO_2]$ conditions (error bars, s.e.m.; n = 10). Letters indicate similarity based on a p-value of 0.05 for independent pair-wise comparisons.

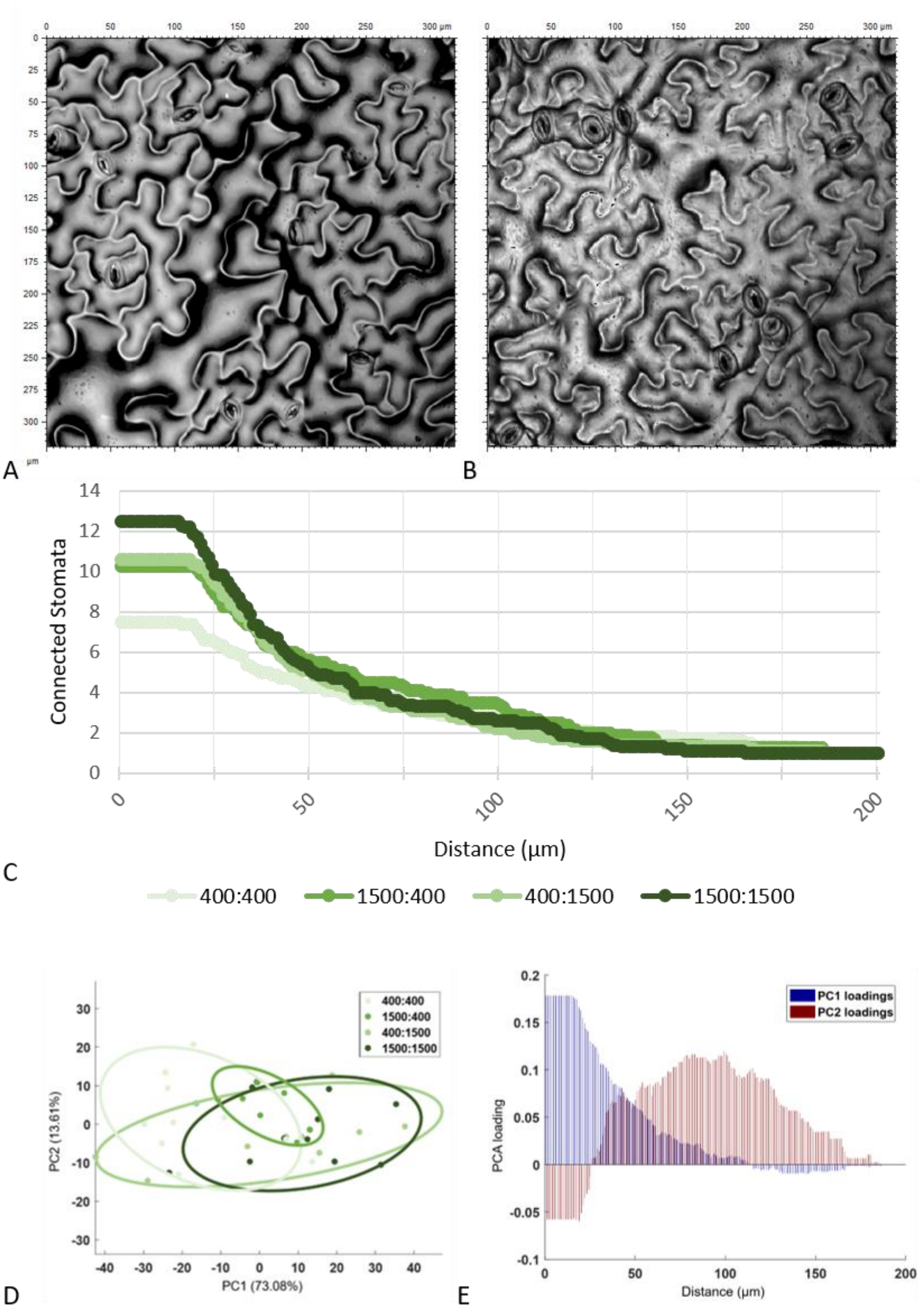

Supplementary Figure 2. Epidermal Patterning. A) Example of random stomatal distribution (scale = µm). B) Examples of grouped stomatal distribution (scale = µm). C) Betti0 curves of raw stomatal number. D) PCA accounts for 86.69% of the variation in the first two components. E) PC loadings for the first two components.

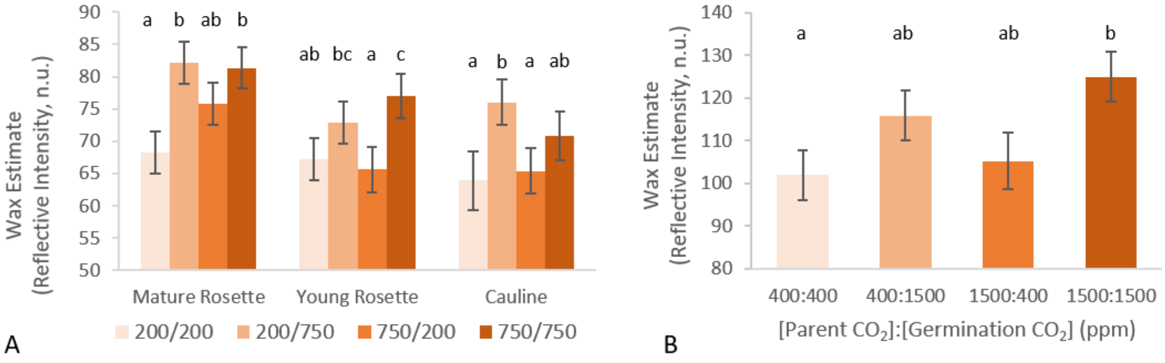

Supplementary Figure 3. Wax deposition is dependent upon the environment experienced by the youngest leaves. A) Three leaf stages of plants grown in a split-environment chamber (error bars, s.e.m.; n = 16) and B) Cotyledons exposed to identical or contrasting parental and germination p[CO_2] conditions. (error bars, s.e.m.; n = 10). Each of the leaf types was analyzed independently and letters do not necessarily correspond across leaf type. Letters indicate similarity based on a p-value of 0.05 for independent pair-wise comparisons.

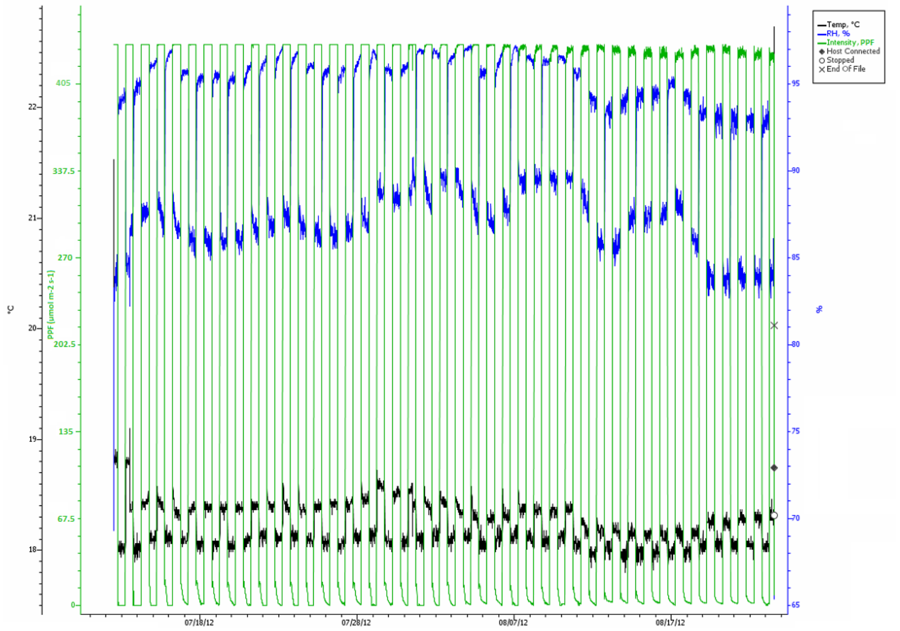

Supplementary Figure 4. Bench-top Chamber Environment. HOBO data logger output from one dual-chamber experimental run. Information shown is representative of all experiments run in dual-chambers.
